# Element banding and organic linings within chamber walls of two benthic foraminifera

**DOI:** 10.1038/s41598-019-40298-y

**Published:** 2019-03-05

**Authors:** E. Geerken, L. J. de Nooijer, A. Roepert, L. Polerecky, H. E. King, G. J. Reichart

**Affiliations:** 1 Department of Ocean Systems, NIOZ-Royal Netherlands Institute for Sea Research, and Utrecht University, Den Burg, The Netherlands; 2 Faculty of Geosciences, Utrecht University, Utrecht, The Netherlands

## Abstract

Trace and minor elements incorporated in foraminiferal shells are among the most used proxies for reconstructing past environmental conditions. A prominent issue concerning these proxies is that the inter-specimen variability in element composition is often considerably larger than the variability associated with the environmental conditions for which the proxy is used. Within a shell of an individual specimen the trace and minor elements are distributed in the form of bands of higher and lower concentrations. It has been hypothesized that differences in specimen-specific element banding patterns cause the inter-specimen and inter-species variability observed in average element composition, thereby reducing the reliability of proxies. To test this hypothesis, we compared spatial distributions of Mg, Na, Sr, K, S, P and N within chamber walls of two benthic foraminiferal species (*Amphistegina lessonii* and *Ammonia tepida*) with largely different average Mg content. For both species the selected specimens were grown at different temperatures and salinities to additionally assess how these parameters influence the element concentrations within the shell wall. Our results show that Mg, Na, Sr and K are co-located within shells, and occur in bands that coincide with organic linings but extend further into the calcite lamella. Changes in temperature or salinity modulate the element-banding pattern as a whole, with peak and trough heights co-varying rather than independently affected by these two environmental parameters. This means that independent changes in peak or trough height do not explain differences in average El/Ca between specimens. These results are used to evaluate and synthesize models of underlying mechanisms responsible for trace and minor element partitioning during calcification in foraminifera.

## Introduction

Element incorporation into foraminiferal calcite provides a valuable tool for reconstructing seawater variables. For instance, foraminiferal Mg/Ca is a well-established proxy for seawater temperature^1–4^, whereas Na/Ca has recently been shown to reflect seawater salinity^5–8^ and Ca-variability^9^. Foraminiferal calibration studies show that a suite of other trace elements (e.g. U, Sr, Li and B), as well as fractionation of certain stable isotopes (e.g. δ^11^B), are related to parameters of the seawater carbonate system^10–16^, whereas Mn incorporation has been suggested to increase with decreasing bottom water oxygenation^17–21^.

Comparison of these foraminiferal calibration studies with inorganic precipitation experiments^22–25^ shows that Element/Ca ratios (El/Ca) and the isotope composition of these elements in foraminiferal shells differ from those in inorganically precipitated calcite. In addition, there is considerable inter-species^26–29^, inter-specimen^30,31^ and intra-shell^32–36^ variability in El/Ca ratios. The underlying biological mechanisms responsible for this variability and for the offset from inorganic-derived partition coefficients are not fully understood^26,37–39^. The proposed mechanisms include selective ion pumping^26,40^, pH regulation^41–43^, seawater vacuoles^26^, involvement of organic matrices^44^, varying precipitation rates^37,45^ and micro-environment effects^46–48^.

A key feature of foraminiferal geochemistry is systematic intra-shell chemical heterogeneity, which has the potential to inform us about the underlying mechanisms of shell formation. This variability is most characteristically manifested in so-called banding, i.e., alternation of high and low concentrations of a given minor or trace element in the direction parallel to the surface of the shell wall^33–35,49,50^. Variability in the patterns of Mg banding, which is present in all species studied, has been hypothesized to be responsible for inter-specimen variability in Mg/Ca^35,36,49,51^. Furthermore, Mg banding is suggested to affect the reliability of the Mg/Ca-based paleo-thermometer, as differences in cleaning procedures between studies might alter the ratio between the amount of bands with high and low Mg concentrations and, thereby, the average Mg/Ca^49^. The biological processes inducing Mg banding are hypothesized to be caused by temporal changes in the chemistry of the foraminiferal micro-environment during calcification. For example, Mg banding has been hypothesized to result from (i) diurnal oscillations in pH in the foraminiferal micro-environment as a function of symbiont activity, which is assumed to have a kinetic effect on Mg incorporation^2,32^, (ii) day-night patterns due to mitochondrial sequestration of Mg^35^, or from (iii) alternating calcification pathways during chamber formation^26,37^. These factors may well be interacting and their relative contribution may vary between species, possibly explaining inter-species differences in Mg banding and average Mg/Ca values.

In addition to Mg, within-wall banding has also been observed for other elements, including Ba and Sr^33^, Na^8,44^, S^34,52^ and B^53,54^. Variation in banding patterns for these elements may explain differences in average El/Ca values between specimens and species, although systematic studies of element banding in foraminiferal shells are scarce. Investigating how banding patterns differ between species and specimens grown at different conditions could enhance our understanding of the dependency of element incorporation on the environmental parameters. Furthermore, comparing element banding between different species and specimens could provide unique insights into the biological controls on element partitioning.

Here we systematically study the relationship between banding patterns and average El/Ca ratios in foraminiferal shells. Specifically, we test the hypothesis that differences in average El/Ca between specimens or species are due to either correlated or independent changes in El/Ca banding width or intensity across the shell wall. To this end, we used NanoSIMS (Nanoscale Secondary Ion Mass Spectrometry) to map the spatial distribution of Mg, Na, Sr and K in shells of two foraminiferal species with distinct Mg-content, *Amphistegina lessonii* and *Ammonia tepida* (~33 and ~3 mmol/mol Mg/Ca respectively^8^) grown under a range of salinities and temperatures. Although these two species are rarely used in paleoceanographic reconstructions, their Mg/Ca-temperature sensitivities and absolute Mg/Ca span the complete range of sensitivities and absolute Mg/Ca known for Rotaliid species (Toyofuku *et al*., 2011; Wit *et al*., 2012). Investigating species that occupy the lower and upper ends of the spectrum increases the possibility to translate the results obtained here to other, paleoceanographically more relevant, species. However, not all species may follow the same ontogenetic patterns as the species studied here, whereby new calcite lamella are added over previous whorls with every new chamber, yet some (e.g. *O. universa, N. dutertrei*) do show daily calcite growth bands, apparently lacking organic linings in between^36^. Therefore, our additional aim is to investigate the spatial structure of the Primary Organic Sheet (POS) and of the subsequent organic linings, and specifically their relationship with the concentrations of Mg, Na, Sr and K across the shell wall. To this end, we used NanoSIMS to additionally measure the distribution of elements primarily associated with organic linings^55^, including N, S and P.

## Methods

### Sample selection and preparation

Specimens of *Amphistegina lessonii* and *Ammonia tepida* measured in this study were selected from previous culturing experiments conducted at different salinities^8^ and temperatures (van Dijk *et al*., in prep). Their average Mg/Ca, Na/Ca and Sr/Ca ratios, derived from measurements by Laser Ablation-Inductively Coupled Plasma-Mass Spectrometry (LA-ICP-MS) on 1–3 chambers, are listed in Table 1.

**Table 1:** Culture conditions and the corresponding single-specimen LA-ICP-MS obtained El/Ca ratios (mean ± SD) in the studied foraminiferal specimens.

Specimen code	Species	Salinity	Temperature (°C)	Na/Ca (mmol/mol)	Mg/Ca (mmol/mol)	Sr/Ca (mmol/mol)	n
1	*A. lessonii*	35	22	7.7 ± 1.13	18.23 ± 1.27	1.42 ± 0.07	3
2	*A. lessonii*	35	29	8.12 ± 0.75	28.07 ± 3.18	1.73 ± 0.22	3
3	*A. lessonii*	35	29	8.45 ± 1.12	34.57 ± 2.49	1.75 ± 0.13	3
4	*A. lessonii*	30	25	7.69 ± 0.2	29.5 ± 0.99	1.86 ± 0.04	2
5	*A. lessonii*	40	25	9.56 ± 0.45	35.93 ± 4.28	1.67 ± 0.07	3
6	*A. lessonii*	40	25	9.8	29.2	1.81	1
7	*A. tepida*	25	25	2.94 ± 0.04	2.07 ± 0.18	1.18 ± 0.04	3
8	*A. tepida*	25	25	3.48 ± 0.03	2.04 ± 0.12	1.26 ± 0.05	2
9	*A. tepida*	35	25	3.90 ± 0.22	1.50 ± 0.21	1.45 ± 0.05	2
10	*A. tepida*	40	25	4.29 ± 0.32	3.39 ± 0.27	1.40 ± 0.03	2
11	*A. tepida*	40	25	4.19 ± 0.13	2.71 ± 0.27	1.41 ± 0.06	3

After the LA-ICP-MS measurements, the specimens were embedded in an epoxy resin (Araldite 2020) in discs of 1 cm in diameter and 5 mm in height. This was done in a vacuum chamber to minimize entrapment of air bubbles within the foraminiferal chambers. After curing for 24 hours at 50 °C, specimens were polished using silicon carbide wet grinding papers with decreasing coarseness (HERMES, WS Flex 18 C, 230 mm, P 800 and 219 ATM, SIC wet grinding paper, grain 4000). This resulted in a cross-sectioned sample with chamber walls exposed perpendicular to the shell walls, as evaluated by light microscopy. Exposed cross sections were subsequently fine-polished using agglomerated alpha alumina powder (Struers AP-A powder, grain size 0.3 µm) and SiO_2_ powder (Logitech SF1 Polishing Suspension, grain size 0.035 µm). Finally, the polished samples were ultrasonically cleaned with ethanol, and coated with a 20 nm gold layer using a sputter coater (JEOL JFC-2300HR high resolution fine coater and JEOL FC-TM20 thickness controller).

### Imaging by SEM, AFM and NanoSIMS

Imaging analyses by scanning electron microscopy (SEM), atomic force microscopy (AFM) and nanometer-scale secondary ion mass spectrometry (NanoSIMS) were performed at Utrecht University. SEM images were taken to identify areas suitable for NanoSIMS analysis and to locate organic linings, while AFM was used to analyze the sample surface topography after the NanoSIMS analysis.

SEM imaging was done with a JEOL Neoscope II JCM-6000 instrument using a backscattered electron detector. AFM imaging was performed with a Bruker Multimode III instrument operating in contact mode using a silicon tip attached to a triangular gold-coated silicon nitride cantilever (spring constant 0.35 N/m, model SNL-10, Bruker). Both height and deflection images were collected, and the measurements were done on the same areas as those measured by NanoSIMS and SEM. Images were taken with a scan rate of 4.15 Hz with 384 lines per image, and the instrument was calibrated prior to imaging using a standard grid with 200 nm pitch resulting in the precision in measured height of 2 nm.

NanoSIMS analysis was performed with the CAMECA NanoSIMS 50 L instrument. Using an element standard (SPI Supplies, 02757-AB 59 Metals & Minerals Standard), magnetic field and exact positions of the electron multiplier detectors were adjusted to enable detection of either positive (^23^Na^+^, ^24^Mg^+^, ^39^K^+^, ^44^Ca^+^, ^88^Sr^+^) or negative (^12^C^−^, ^16^O^−^, ^12^C^14^N^−^, ^31^P^−^, ^32^S^−^) secondary ions. The positive and negative secondary ions were detected using an 8 keV primary O^−^ and Cs^+^ ion source, respectively. First, areas of interest were pre-sputtered until secondary ion counts stabilized. The same pre-sputtering protocol was used on all samples to ensure comparability between different fields of view (FOV) measured. Subsequently, ion count images were acquired by rastering the primary beam over the sample surface (areas between 8 × 8 and 40 × 40 µm in size) using the following diaphragm and slit settings: D0–2, D1–3, ES-2, AS-0 and EnS-0 for the O^−^ beam, and D0–0, D1–3, ES-2, AS-2 and EnS-1 for the Cs^+^ beam. These settings yielded a primary O^−^ beam at the sample surface of ~2 pA with a nominal size of 300–500 nm (defined by CAMECA) and a primary Cs^+^ beam of ~0.5 pA with a nominal size of ~100 nm, as certified by CAMECA for the NanoSIMS instrument used. Additionally, the settings gave sufficient mass resolution to separate isobaric interferences while keeping high transmission. Secondary ions were detected with a dwell time of 2 ms/pixel (positive ions) and 0.8 ms/pixel (negative ions). To increase the overall signal the same FOV was imaged multiple times (400–1000), and the resulting ion count images were aligned and accumulated.

### Data processing and analysis

Data processing was done using the freeware program Look@NanoSIMS^56^ as well as additional custom-made routines in Matlab. First, effects of sample surface topography on the measured variability of elements within shells were assessed by inspecting the overlay between the AFM and NanoSIMS images. Second, the spatial correlations between the organic sheets and the banding patterns seen in the distribution of elements were verified by overlaying the SEM and NanoSIMS images. Lateral profiles were drawn from the aligned SEM and NanoSIMS maps to allow comparison of the position of the organic linings.

The NanoSIMS images were aligned with the corresponding AFM or SEM images by manually adjusting the relative angle, displacement and magnification of one image against the other, which was done using a new interactive alignment tool added to Look@NanoSIMS (Fig. S1). To ensure unbiased alignment the adjustments were made based on non-chemical features seen in the images, such as pores and edges of the calcite shell. Furthermore, this alignment strategy was compared with alignment of Ca-maps to SEM images of the square-shaped crater created by the NanoSIMS analysis, which resulted in an indistinguishable alignment fundamentally independent from the first approach.

Further analysis involved lateral profiles of El/Ca (for positive ions) and El/O (for negative ions) ratios along lines oriented perpendicularly to the banding defined by organic sheets. When defining the lines, pixels close to edges, cracks or pores within the shells were avoided. Ten lateral profiles along lines parallel to the defined line were averaged to improve signal-to-noise ratio.

For each specimen, El/Ca ion count ratios obtained from the lateral profile were averaged and plotted against the corresponding average molar El/Ca ratio derived from the LA-ICP-MS analysis. Since the two data sets showed good linear correlation when all specimens were combined (Table 2 and Supplementary Table 1, Fig. S2), the regressions were used as calibration lines to convert the NanoSIMS-derived ion count ratios to molar El/Ca ratios in every pixel of the image.

**Table 2:** Results of linear regressions between the averaged ion count ratios obtained from NanoSIMS lateral profiles and the average molar El/Ca ratios obtained by LA-ICP-MS (Table 1).

El/Ca	Slope	SE_slope	tStat	Intercept	R^2^	p-Value
log(Mg/Ca)	0.91	0.06	14.77	−1.47	0.92	1.67*10^−11^
Na/Ca	2.34*10^−1^	2.45*10^−2^	9.54		0.27	1.11*10^−8^
Sr/Ca	2.38*10^−2^	6.86*10^−4^	34.68		0.46	1.21*10^−18^

Mg/Ca were log-transformed for both the NanoSIMS and LA-ICP-MS data to account for the order of magnitude difference in Mg content between the two studied species. Na/Ca and Sr/Ca relationships were forced through zero. Note that the regressions are subject to some uncertainty as the areas analyzed by NanoSIMS were on different chambers than those ablated and analyzed by LA-ICP-MS.

**Table 3:** Orthogonal regression results of lateral profiles for Mg-Na, Mg-Sr and K-Na for *A. tepida* and *A. lessonii* specimens grown under different experimental conditions.

*A. lessonii*	Experimental condition	Slope	Intercept	R	SD Slope	SD Intercept
Mg-Na	Sal 30	0.67	0.34	0.93	4.46*10^−2^	4.12*10^−2^
	Sal 40	1.56	0.73	0.71	1.43*10^−1^	8.07*10^−2^
	Temp 22	1.63	1.22	0.79	1.33*10^−1^	9.09*10^−2^
	Temp 29	1.74	1.04	0.83	1.38*10^−1^	1.51*10^−1^
Mg-Sr	Sal 30	0.02	0.03	0.69	2.69*10^−3^	2.25*10^−3^
	Sal 40	0.03	0.02	0.45	3.96*10^−3^	2.20*10^−3^
	Temp 22	0.02	0.03	0.56	2.81*10^−3^	1.83*10^−3^
	Temp 29	0.02	0.02	0.54	2.95*10^−3^	3.18*10^−3^
K-Na	Sal 30	36.56	0.31	0.74	3.95	6.70*10^−2^
	Sal 40	14.16	0.95	0.54	1.46	7.09*10^−2^
	Temp 22	23.25	1.15	0.85	2.20	9.68*10^−2^
	Temp 29	11.70	1.57	0.74	1.23	1.26*10^−1^
***A. tepida***	**Experimental condition**	**Slope**	**Intercept**	**R**	**SD Slope**	**SD Intercept**
Mg-Na	Sal 25	8.40	0.18	0.67	1.36	8.40*10^−2^
	Sal 35	11.87	0.29	0.81	1.41	1.01*10^−1^
	Sal 40	14.08	0.76	0.56	2.28	1.79*10^−1^
Mg-Sr	Sal 25	0.23	0.02	0.59	4.31*10^−2^	2.16*10^−3^
	Sal 35	0.27	0.02	0.33	5.39*10^−2^	3.74*10^−3^
	Sal 40	0.22	0.02	0.47	4.05*10^−2^	3.55*10^−3^
K-Na	Sal 25	20.57	0.37	0.65	3.28	5.30*10^−2^
	Sal 35	1.10	0.78	0.88	9.52*10^−2^	3.08*10^−2^
	Sal 40	2.9	1.27	0.77	3.23*10^−1^	7.81*10^−2^

*Sal* stands for salinity experiment and *Temp* for temperature experiment, see Table 1 for details. See Fig. S6 for scatter plots and regression lines.

A typical lateral profile of El/Ca ratios within the foraminiferal shells comprised a succession of peaks and troughs. To allow comparison between specimens or different locations within the same specimen, we converted each profile to a corresponding density distribution derived from the histogram of the El/Ca values within the profile. Additionally, we reduced each profile to a set of descriptors that included peak locations, peak width and height, and trough height. This was done in Matlab using a local peak finder function, which locates peaks for data points higher than its two neighbors and determines the peak prominence, i.e. the difference between the peak height and the two lowest neighboring values. A minimal acceptable peak prominence was set to avoid identification of noise in the data as false peaks. Peak and trough heights were defined, respectively, as the average height of the peaks and troughs in the profiles.

The effect of the size of the NanoSIMS primary ion beam on the shape and width of the peaks in the lateral profiles of El/Ca and El/O ratios was assessed by calculating the spatial convolution between the Gaussian function, which represented the shape of the NanoSIMS beam, and an idealized distribution of the organic elements within the shell represented by a top-hat function^43^. The width of the NanoSIMS beam was derived from the nominal beam size for the given instrument settings (full width half maximum (FWHM) of 355–640 nm for the O^−^ and 100 nm for the Cs^+^ beam), whereas the width of the top-hat function was assumed to be equal to the thickness of the organic linings derived from the SEM images (270 nm).

## Results

### Intra-shell element banding and organic linings

**Figure 1:**
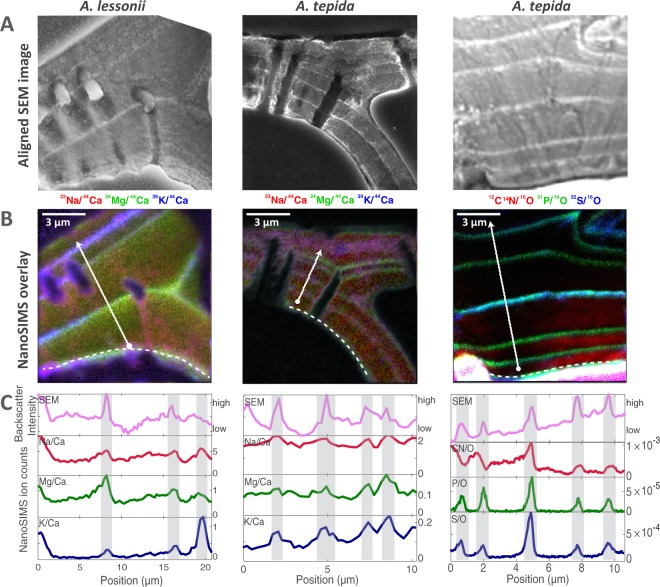
Comparison of backscattered electron images obtained with SEM and NanoSIMS RGB maps. (**A**) Backscattered electron images, aligned to the NanoSIMS maps, showing the polished cross-sections of one specimen of *Amphistegina lessonii* (left column) and two of *Ammonia tepida* (middle and right) embedded in resin. (**B**) NanoSIMS RGB maps showing spatial distributions of El/Ca in *A. lessonii* (left), *A. tepida* (middle) and El/O in *A. tepida* (right), corresponding to specimens #5, #9 and #11 (Fig. 3 and Table 1). Dashed white lines indicate the position of the Primary Organic Sheet, which appear similar in intensity to the other organic linings in the shell. (**C**) Lateral profiles (white arrow indicated in panel B) through the aligned SEM images and NanoSIMS maps, showing the overlap between the brighter lines of the SEM image and peaks in high El/Ca and El/O.

**Figure 2:**
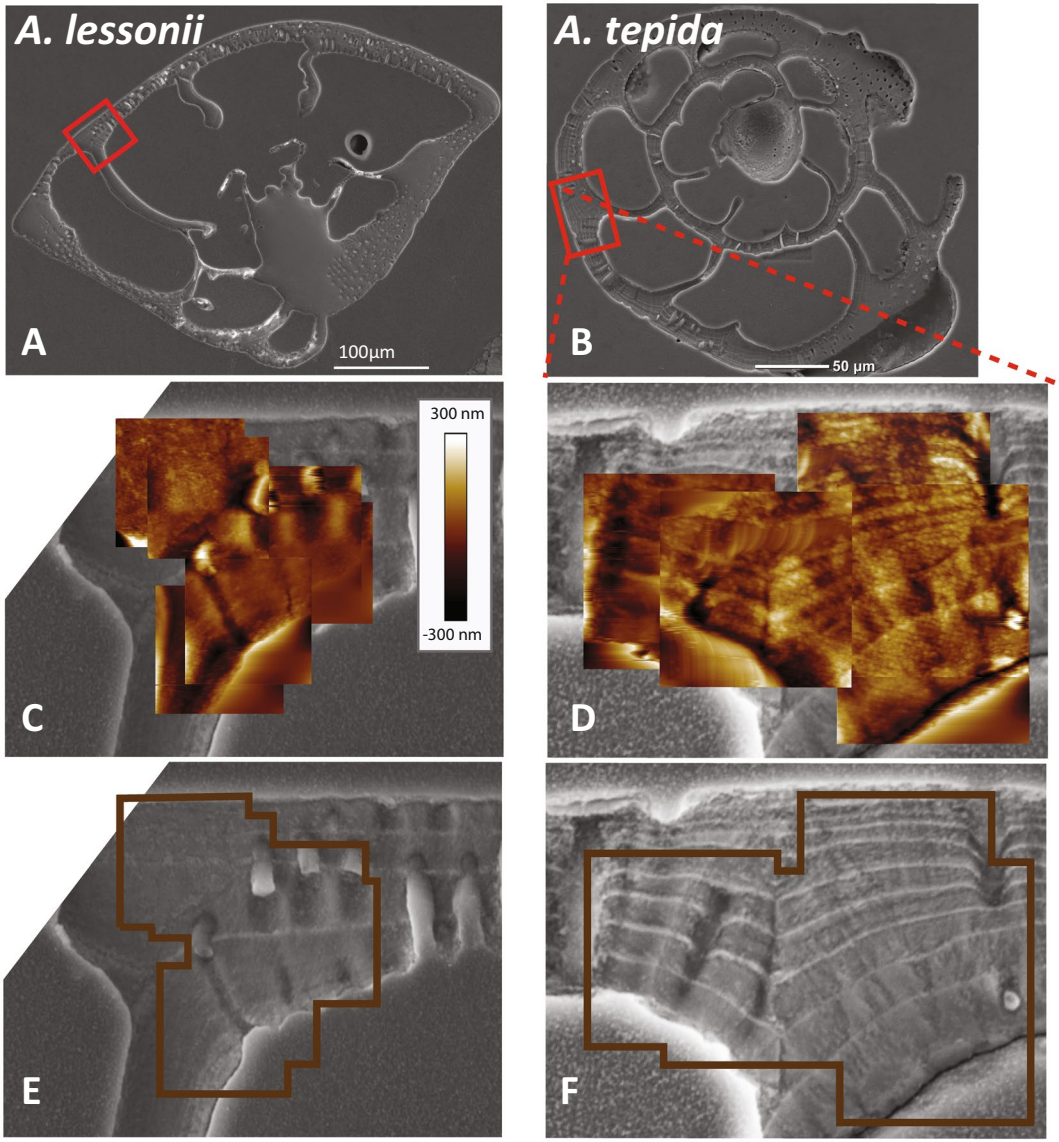
Overview SEM images of two specimens (**A**,**B**) and AFM height images superimposed on a close-up backscattered electron image (**C**,**D**), showing more pronounced topography related to organic linings in a specimen of *A. tepida* (**A**,**C**) compared to that in *A. lessonii* (**B**,**D**). The underlying SEM images show the lamellae typical of Rotalid species that are responsible for the topography (**E**,**F**) with the outline of the AFM maps from **C**,**D**). The color scale bar is the same for both species. Note that there are some distortions in the *A. tepida* specimen that do not reflect height.

All SEM images of *A. lessonii* and *A. tepida* shells show thin brighter bands between calcite lamellae (e.g. Figs 1 and 2). These bands have been previously identified as organic linings^55,57,58^, and our NanoSIMS data corroborate this interpretation. Specifically, images of elements associated with organics (N and P) show thin bands at the same positions as the brighter bands in the SEM images (Fig. 1). Similar bands are also visible in images of S (Fig. 1), but this element can be associated with both organics^55^ and calcite^34^. While S bands are pronounced in shells of both species, N and P bands are more pronounced in *A. tepida* (Fig. S3). This difference between species is reflected also in the SEM images, which show brighter, thicker and hence more clearly visible bands for *A. tepida* (Fig. 1).

**Figure 3:**
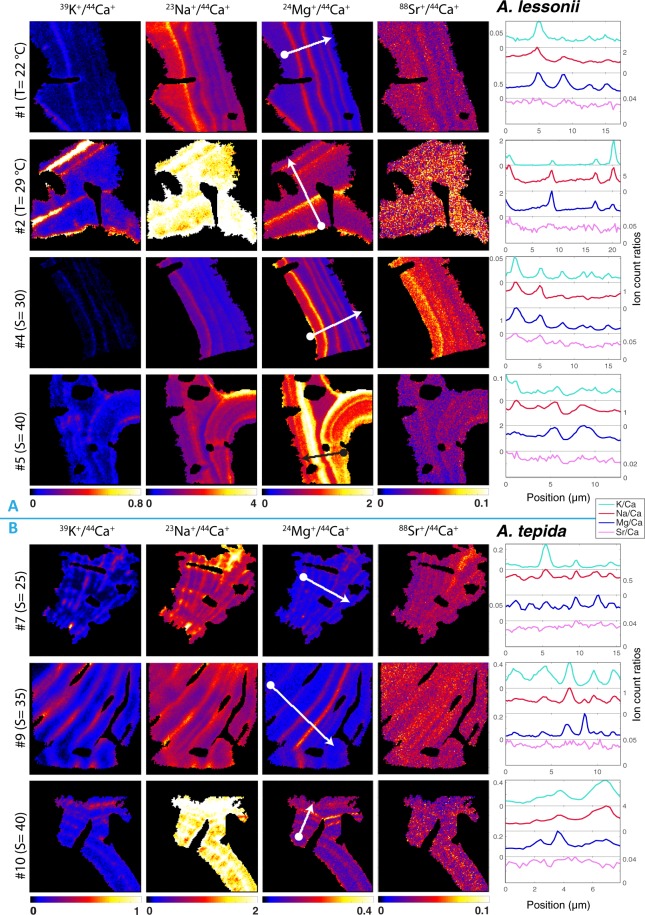
Examples of representative NanoSIMS count ratio maps of K/Ca, Na/Ca, Mg/Ca and Sr/Ca (left to right) of *Amphistegina lessonii* specimens #1, #2, #4 and #5 (panel A) and *Ammonia tepida* specimens #7, #9 and #10 (panel B) for the salinity experiment (S = 25, S = 30, S = 35, and S = 40) and temperature experiment (T = 22 °C and T = 29 °C). Specimen numbers correspond to specimen ID in Table 1. Color map scales of El/Ca maps are the same between specimens to facilitate comparison between maps and measured LA-ICP-MS El/Ca values. Lateral profiles (right panel) show that El/Ca peaks generally coincide and are asymmetrical in *A lessonii*. Profiles, with a width of 10 pixels, are indicated with white arrows showing the direction from inside to outside of the shell wall (Mg/Ca panel).

For both species the distributions of Mg, Na and K show clear banding patterns (Fig. 3). The banding patterns are similar to those observed previously in samples of the same species by an electron microprobe^59^. Close inspection of the overlays between the NanoSIMS and SEM images and of the corresponding lateral profiles shows that the peaks of these metals are found at the location of the organic linings (Fig. 1). Thus Mg, Na and K are spatially linked with the organic linings in the shells of the studied species. Sr is more homogeneously distributed than the other metals, however it does seem to show a banding pattern as well (Fig. 3). Analysis of all available SEM and NanoSIMS image pairs revealed no apparent systematic differences when comparing the primary organic sheet (POS) with subsequent organic linings. Therefore we henceforth refer to them collectively as ‘organic linings’.

AFM measurements show that the NanoSIMS analysis affected the surface topography of the polished shells. For example, for both species the variability in the surface height clearly increased around the pores and shell edges (Fig. 2). Additionally, organic linings in the shells of *A. tepida* became more elevated than the calcite lamellae positioned between them, whereas the linings were not clearly distinguishable in the AFM images of *A. lessonii* (Fig. 2). In NanoSIMS measurements it is sometimes observed that a variation in the surface topography (e.g., around edges) affects the intensity of the secondary ion counts. That this “edge effect” was relevant in our measurements is illustrated by the pronounced increase in the El/Ca ratios around the pores and shell edges (Fig. S4). However, if it also played a role around the organic linings, which were, for *A. tepida*, elevated relative to the surrounding calcite lamellae and thus had edges on both sides, one would expect to observe elevated El/Ca ratios on both sides of the organic linings. Similarly, if the “edge effect” were important, one would expect to observe less distinct El/Ca bands around the organic linings of *A. lessonii* because of their less pronounced topography variation. Since both of these expectations are not supported by our data (Fig. S4), we conclude that the El/Ca peaks observed around the organic linings are real and not due to an analytical artifact.

Although spatially linked, there is a systematic difference between the bands of the “organic elements” (N, P and S) and the studied metals. While the widths of the organic element bands are about 280 ± 50 nm, the metal bands are significantly broader (810 ± 160 nm for *A. tepida* and 1160 ± 280 nm for *A. lessonii*). Numerical analysis revealed that this difference cannot be explained by the larger size of the O^−^ beam used for the measurements of the metals in comparison to the Cs^+^ beam used for the measurements of the organic elements (Fig. S5). Thus we conclude that the metal peaks in the studied shells are broader than the organic linings. The widths of the organic element bands are about 2–3 fold larger than the previously reported values (100–130 nm^44^), but this difference is likely due to the presence of a nanometer-scale branched organic 3D-structure surrounding the sheet^44^, falling within this width.

### Intra-shell element distribution: variability between species and specimens

Although the two species studied here have about 10-fold different average Mg/Ca ratios, their element banding patterns are generally similar. In both species, metal bands are parallel to the inner- and outer surface of the chamber walls, following the lamellar structure of Rotaliid chamber formation. The main difference between *A. tepida and A. lessonii* is that both peaks and troughs for Mg/Ca are about 10-fold higher (Fig. 3), and peaks are about 1.4 broader, in *A. lessonii* compared to *A. tepida* (Fig. S5). Furthermore, relationships between Na/Ca and Sr/Ca versus Mg/Ca along the lateral profiles show steeper slopes in *A. tepida* (Table 3, Fig. S6). Additionally, metal peaks in *A. lessonii* are more asymmetrical, increasing towards the outside of the lamella in most maps (e.g., specimens #2 and #4) while declining towards the outside in others (e.g., specimen #1), especially for Na/Ca (Fig. 3). In contrast, metal peaks in *A. tepida* are more symmetrical and narrower, hence more closely confined to the organic linings separating the lamellae, especially for K/Ca (Fig. 3).

**Figure 4:**
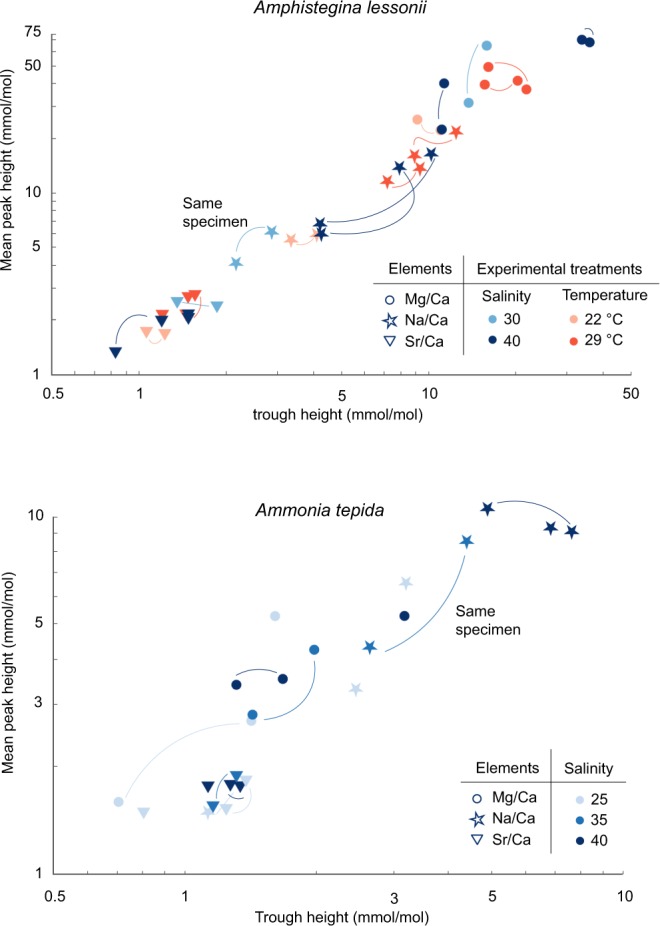
Average peak height versus trough height for Mg/Ca (circles), Na/Ca (stars) and Sr/Ca (triangles) based on lateral profiles (see Supplementary Table 2 for regression results) for *A. lessonii* (top) and *A. tepida* (lower panel). Every symbol represents one NanoSIMS map: two maps from the same specimen are connected with a line. Both peak and trough values increase (proportionally) with increasing average El/Ca values, resulting either from environmental effects (e.g. higher Mg/Ca with increasing temperature) or inter-specimen variability (grown in the same environmental conditions yet different average El/Ca values).

Both the NanoSIMS and LA-ICP-MS data^8^ show that there is considerable variability between specimens with respect to their El/Ca lateral profile and average El/Ca values, irrespective whether they have been grown in different or the same environmental conditions. Interestingly, an increase in the average El/Ca ratio, as measured by LA-ICP-MS, is related to an increase of both peak and trough height of the lateral profiles, as measured by NanoSIMS (Figs 4 and S2). In contrast, peak widths do not show a significant relationship with the mean El/Ca values. In both species the distance between peaks is determined by the width of the lamella, which increases with chamber number. Peak distance would therefore systematically affect inter-specimen differences in El/Ca only if lamellae became thinner with higher El/Ca, which is not observed. Together, these observations show that inter-specimen variability, between specimens from the same or different conditions, is not explained by independent changes of the peak or trough height, but is driven by a proportional increase of the entire profile (Figs 4 and S2).

For the *A. lessonii* specimens studied here, increased average Mg/Ca, Na/Ca and Sr/Ca in specimens grown at a higher temperature or higher salinity results from a proportional increase of both peaks and trough heights (Fig. 4). It has to be stressed, however, that our study does not reflect the response to environmental conditions at a population level, and we refer to van Dijk *et al*. (Biogeosciences, in prep.) for results showing temperature effect on Sr/Ca, Mg/Ca and absence of an effect of temperature on Na/Ca. Our study shows, however, that El/Ca peak heights are ~2 times higher than El/Ca trough heights (see Supplementary Table 2 for regression results), and that the entire within-shell element distribution of an *A. lessonii* specimen is thus elevated with increasing average-shell El/Ca values (Figs 4 and S2). For *A. tepida*, El/Ca peak height shows a significant linear relationship with El/Ca trough height, with a slope of 1.4 and intercept of 0.7 (see Supplementary Table 2 for regression results). Both Na/Ca peak and trough height are increased in specimens grown at higher salinities, however, the trend is not fully proportional, since the peak height intercept is positive (Fig. 4).

## Discussion

### The role of organic linings in calcification and the relationship with element banding

Organic linings play a fundamental role during the first phase of carbonate precipitation in foraminifera, by providing a template upon which calcification occurs^57^. However, the organic template of many organisms (e.g. oysters, mollusks) likely provides more than just structural support^58,60,61^. Organic linings in foraminiferal shells have been shown to be spatially^32^ or even mechanistically associated to trace and minor element bands in some species, where the primary organic sheet (POS) is suggested to adsorb cations^43^. This POS is formed by a dense pseudopodial network prior to calcification, and gives the new chamber its overall shape^48,49^. Furthermore, organic linings are suggested to have a branched structure in *O. universa* rather than a laminar sheet structure^43^. Organic linings differ in composition and characteristics between species, with *A. tepida* showing organic linings that are more resistant to decalcification compared to those of other species^58^. This is in line with our SEM and AFM results, showing brighter and more elevated organic linings in *A. tepida* than in *A. lessonii* (Figs 1, 2). Furthermore, this difference in the organic linings of both species is also reflected in the NanoSIMS maps, which show more pronounced P and N peaks in *A. tepida* than in *A. lessonii* (Fig. S5). Possibly, these different characteristics of the organic linings are related to the difference in Mg/Ca content between species, for example by affecting Mg uptake or by controlling nucleation (e.g. by determining the CaCO_3_ phase formed).

Elevated signals of N, P and S in our NanoSIMS maps (Figs 1 and S5) likely originate from organic compounds such as proteins and sulfated polysaccharides that are present in organic linings of foraminifera^55^. Sulfated and acidic groups of these compounds are hypothesized to help overcome the free energy barrier prohibiting crystal nucleation and guide crystal growth^24,55,61^. P peaks are broader (~270 nm) then previous estimations of organic lining thickness (~100–130 nm^44^), which could be due to the presence of a smaller-scale 3D structure within the P peak width. These structures have been observed in *O. universa* with Transmission Electron Microscopy^44^, yet cannot be resolved within the spatial resolution of our Cs-source maps (~100 nm). Within the calcite lamella, P ion counts are low (between 0–2 counts, 100 times lower than in the organic linings), suggesting that the organic linings and a surrounding small scale 3D structure around the sheets of *A. tepida* and *A. lessonii* is confined to a small region (~300 nm) rather than a substantial branched structure extending into the calcite lamella (~5 μm). However, since P ion counts do not entirely decline to zero (1–2 counts), single organic macromolecules might be present at small concentrations, within the calcite or between the calcite rods, as we cannot detect how ions are incorporated. Single organic macromolecules are observed in other biomineralizing species such as mollusks^62^, and have been suggested to increase the hardness of the carbonate shells^63^.

Sulfur peaks observed in our NanoSIMS maps can be indicative of the sulfated groups of the organic compounds that form the organic linings^55^. Although S-peak widths are similar to P-peak widths (both ~270 nm), S counts are ~10 times higher than P counts, and S counts do not decline to zero in between the peaks (10–15 counts, Figs 1 and S5). Furthermore, in some specimens S peaks show shoulders (asymmetrical) within the lamella between the organic linings (Figs 1 and S5). This could be sulfur in the form of calcite-bound SO_4_^2−^ ^34^, yet our study cannot resolve between calcite or organic hosted sulfur. Furthermore, organic-bound elements potentially have different ionization efficiencies, making it difficult to directly compare relative S/O-ion counts between the organic and calcite fractions. However, previous studies suggest that the calcite-bound SO_4_^2−^ fraction is higher than the organic-bound S fraction^34,52^.

Our finding that El/Ca peaks are co-located with organic linings (Fig. 1) is in line with other studies on intra-specimen element distribution in Rotallid (benthic) foraminifera^8,33,37,52,64^. An Atom Probe Tomography-Time-of-flight-Secondary Ionization Mass Spectrometry study showed that the interface of the organic template in *O. universa* is enriched in Na and Mg^44^, suggesting that metals other than Ca^2+^ might be bound to charged functional groups, or that incorporation of these elements is increased due to lattice strain induced by the organic template. It has been suggested that organic-bound Na contributes significantly to Na-variability within the shell, and hence may affect the paleo-application of this novel proxy^44^. Adsorption to the organic template may well explain part of the observed El/Ca peaks, especially in *A. tepida*, showing peaks that are closely positioned around the organic linings. However, our measurements show that the El/Ca peaks, particularly in *A. lessonii* profiles, are significantly broader than organic elements peaks (Fig. S3). Furthermore, a recent study using X-ray spectroscopy demonstrated that part of the Na is most likely calcite-bound in foraminifera shells^65^. This is corroborated by the relatively high Na/Ca-troughs in our NanoSIMS maps (Fig. 3), suggesting that there is considerable Na-variability in both *A. tepida* and *A. lessonii* calcite, which is not associated to high levels of organic compounds. However this study cannot resolve whether Na is structurally bound in the calcite or present between the rods. K/Ca peaks appear to be more strictly confined to the organic template and decline to near zero values in the lamella (Fig. 3), resembling the spatial distribution of organic elements. Since metal peaks are broader than the organic element peaks, and peak heights are related to trough heights for all elements, the calcification mechanism employed by foraminifera might play a role in the banding patterns, which is discussed in more detail in section 4.3.

### Environmental effects on element banding and inter-specimen variability; implications for proxies

The first reports on intra-shell Mg heterogeneity^32,66^ questioned the reliability of the Mg-paleothermometer. More recent studies have aimed to reconcile this large variability in composition with an overall and consistent increase in Mg/Ca with temperature, for example by showing that both low- and high-Mg concentration bands increase with temperature^35,36^. In our dataset, an increase in average Mg/Ca, both between species and between specimens, regardless of environmental condition, is accompanied by a proportional increase in both Mg/Ca peak and trough height. Mg/Ca peak height is hence systematically related to through height, whereby the relative intra-shell variability remains similar (Fig. 4). NanoSIMS profiles have been used previously to examine Mg/Ca ratios taken from single specimens transferred to higher temperatures, allowing the authors to circumvent potential offsets due to inter-specimen variability^36^. Our increasing Mg/Ca peaks and troughs observed in *A. lessonii* specimens grown at higher temperature (Fig. 4) fit well with the lateral profiles of single specimens in the previous study, indicating that the proportional increase is applicable both within and between specimens and is depending on temperature. However, in contrast to our findings, a study using depth-resolved LA-ICP-MS measurements shows that a similar, rather than a proportional, increase in both low- and high-concentration Mg-bands, occurs in specimens of *O. universa* grown at increasing temperatures^35^. This difference may result from differences between species (or groups: e.g. benthic versus planktic) or between experimental and methodological treatments (e.g. NanoSIMS in this study versus LA-ICP-MS profiles^35^). Temperature seems hence to exert an overall effect on the trough as well as peak Mg concentrations through thermodynamic principles, kinetic effects, affecting the Mg uptake mechanism by the organism or a combination of these effects^67^. Our NanoSIMS analysis and results from previous studies^35,36^ increase confidence in the Mg-paleothermometer, as the overall element’s spatial distribution is systematically raised (Fig. 4). This appears to justify a working model on foraminiferal Mg/Ca based on the observation that Mg is incorporated in a uniform crystallization mechanism^67^. It has to be noted however that the latter study^67^ did not capture the primary organic sheet itself, so part of the Mg peak might still be adsorbed to the organics. Future research should hence look at the incorporation of elements into the calcite in the vicinity of organic templates, within ~300 nm.

The incorporation of Na in foraminiferal shells has been shown to depend on salinity^5,6,8^ and seawater Na/Ca^9^. This could be explained by an effect of ionic strength on the Na activity or partition coefficient^5^. Na/Ca peaks and trough heights increase with increasing salinity, proportionally in *A. lessonii* and almost proportional in *A. tepida* (Figs 3, 4), although the limited number of NanoSIMS maps does not allow for robust statistical testing. The correspondence between the positive peak intercept in *A. tepida* (Fig. 4) for Mg and Na with the organic linings may be explained by a relative large contribution of adsorbed Mg and Na expected in the organic linings^44^. This might influence the paleo-application of Na/Ca to reconstruct salinity, since degradation of the organic linings and adsorbed Na might alter the average Na/Ca over time^44^. Furthermore, it has been suggested that also structurally bound Na is leached due to burial diagenesis^65^, which further complicates the down-core application of Na/Ca.

Sr/Ca banding in foraminiferal shell walls is generally less pronounced than that of Mg/Ca, K/Ca or Na/Ca (Fig. 3). Subtle changes in Sr/Ca were also observed within the test wall of *Pulleniatina obliquiloculata*, with high ion counts coinciding with the high Mg-bands^33^. The fact that Sr/Ca banding is (much) less pronounced than that observed for Na/Ca and Mg/Ca may also be explained by the Sr counts being low in both species. In *A. lessonii*, Sr/Ca trough and peak heights increase with temperature (Fig. 4). This is in line with van Dijk *et al*.^59^ showing that, in addition to increasing Mg partition coefficients, Sr partition coefficients also increase with increasing temperature. This suggests that in high-Mg species, Sr concentration is not only affected by a carbonate ion effect^12^ but also by temperature. This effect could be direct or indirect, e.g. through Mg induced differences in lattice strain, which enhances Sr incorporation^45,68^. This is consistent with our results, showing that Mg/Ca and Sr/Ca are strongly correlated along the lateral profiles. In *A. tepida*, Sr/Ca banding is less clearly pronounced (Fig. 3).

The K/Ca ion counts within test walls show a strong correlation with Na/Ca ion counts (Fig. S6, Table 3), and both K/Ca minima and maxima increase with salinity, although K/Ca peaks are highest at salinity 35 for *A. tepida*. Since Na/Ca has been shown to correlate with salinity, a similar within-shell wall behavior of K/Ca suggests that also this element might have the potential for constraining past salinities, although application will depend on a suitable analytical approach to accurately and precisely determine K/Ca.

**Figure 5:**
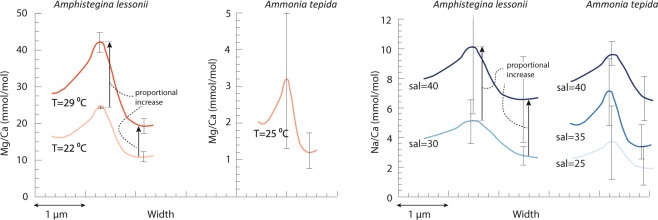
Illustration of the average element banding patterns with environmental conditions, based the average El/Ca peak and trough height per condition, showing the proportional increase in Mg/Ca peak and trough height between species and between *A. lessonii* specimens grown in different temperatures (left). The effect of salinity (right) on Na banding in *A. lessonii* and *A. tepida* is a linear increase of the trough and peak height, whereby the trough is increasing relatively more than the peak height. Error bars indicate +/− 1 SD surrounding the mean peak and mean trough values. The position of the error bars is sometimes offset along the x-axis for clarity and for the Na/Ca of *A. lessonii* grown at a salinity of 40, the +1 SD has been partly removed.

Altogether, these results show that element banding itself, e.g. uncorrelated changes in peak or trough height, does not explain observed differences in El/Ca among specimens as a function of e.g. temperature, salinity or inter-specimen variability (Figs 4, 5 and S2). The simultaneous increase in peak and trough height across elements (Fig. 4) hints to a single mechanism responsible for the intra-shell variability in Mg/Ca, Sr/Ca and Na/Ca, although it remains to be investigated what this control is. Our results confirm that calibrating El/Ca to environmental conditions using LA-ICP-MS data requires careful inspection of the profiles for completeness, e.g. covering the entire thickness of the shell wall.

### Implications for biomineralization

Element banding in foraminiferal shells most likely reflects biomineralization related processes. Banding is most pronounced for elements under tight biological control such as Mg^38,69^. Several biomineralization mechanisms have been invoked to explain the element bands in planktic foraminifera, such as differences in symbiont activity^32,49,70^, changes in Mg flux to control calcite precipitation^71^ or induced by diurnal light-dark cycles^35,36^.

Elevated concentrations of Mg, Na, Sr and K coincide with the position of organic linings, suggesting that incorporation of these elements is relatively high during the initial phase of the formation of a calcite layer or that they are potentially associated with the organic lining. In addition, concentrations of the metals analyzed here in some specimens (e.g. specimen #2) also (gradually) increase towards the outside of an individual lamella, indicating that also the final stages of the formation of an individual calcite lamella are accompanied by a weaker elemental discrimination compared to seawater El/Ca, especially in *A. lessonii*. This observation could be explained by Rayleigh fractionation^72^, whereby elements are incorporated from a biomineralization reservoir with element-specific organic partition coefficients. With ongoing calcification, the Ca-fraction in the reservoir is reduced by precipitation of CaCO_3_ and elements with an organic partition coefficient <1 (such as for Sr, Mg and Na) will hence increase towards the end calcification^72^. However, recent studies suggested that Ca is taken up from the seawater during calcification^39,40^, either through Ca-pumps, channels or vacuoles enriched in Ca, which would (partially) counter-act the Rayleigh fractionation effect. Alternatively, changes in element concentrations within a lamella might also be related to variability in e.g. the precipitation rate^72^, Ca-influx or pH at the site of calcification. In addition, the tight control on leakage of trace element during calcification may decrease towards the end of a calcification event so that the fluid from which calcite is precipitated starts to resemble seawater and foraminiferal partition coefficients are closer to those reported for inorganically precipitated calcite.

Co-location of element bands (^33,34,70^, this study) likely relates to the reported inter-element correlations between species^28,29,45^ and between specimens^8,73^. There are several hypotheses that provide an explanation for co-located element banding that will be discussed to first test their applicability to our and previously published results and, second, to evaluate potential environmental controls (i.e. temperature and salinity) on element incorporation and banding therein.

#### Dual-phase calcification

It was proposed that the formation of every individual lamella is a two-step process: an initial high Mg-phase is precipitated close to the organic lining, followed by an additional low-Mg phase^37^. These two phases are thought to be formed by smaller and larger spherulites, consisting of high- and low-Mg calcite, respectively, as observed in decalcified specimens of *A. lobifera*. Changes in the relative contribution of these high Mg-phases (for instance due to a higher temperature) could be responsible for the observed increase in average Mg/Ca with temperature^26^. Formation of the high-Mg spherulites was further hypothesized to invoke secondary calcification by reducing the Mg concentration within the site of calcification (SOC). These first smaller spherulites would also be enriched in P, which together with Mg^2+^ is the major inhibitor for calcite nucleation and growth in seawater^74^. Such a different crystallographic phase may affect the incorporation of other elements as well, which is in line with the general correlation between all elements observed in our study. Our NanoSIMS data showing El/Ca peaks around the organic linings and troughs in between (Fig. 3) appears to confirm the existence of the suggested different phases^26^. However, we also show that the entire intra-shell El/Ca lateral profiles distribution (both peak and trough height) shifts towards higher El/Ca ion counts for specimens (and species) with higher mean El/Ca values (Fig. 4). This is in contrast to the suggested increase in the relative contribution of the high-Mg phase explaining variability in Mg/Ca between specimens^26,37^. Furthermore, a uniform crystallographic Mg coordination in both high- and low-concentration bands^67^ suggests that the two types of bands only differ in their Mg-content, but are otherwise crystallographically similar. Together, this questions the dual-phase crystallization pathway as the primary mechanism for the observed inter-species variability in Mg/Ca and other El/Ca ratios.

#### Adsorption to organic molecules

Surface adsorption of cations to negatively charged functional groups at the interface of the organic matrix^44^ could explain the observed coupled increase of cations near the organic linings. Observed banding for P and N, which most likely reflects the actual organic material, is confined to the organic linings as observed with SEM, than the broader bands of the metals measured here (Figs 1 and S1). As these smaller metal ions can be accommodated in the calcite structurally, this could explain the gradual decline away from the organic lining (Fig. 3) versus the sharp peaks of organic elements (Fig. S5), probably incorporated as PO_4_ and NO_3_ or amino acids, that are incompatible with the calcite structure. Note that we cannot distinguish between organic and calcite-bound ions, and that there could be a matrix effect, which could produce apparent higher counts during the NanoSIMS analysis. Some of the metals that produce the observed peaks, might hence be adsorbed to organic compounds, which for Na might compromise its use as a salinity proxy^6^. In *A. tepida*, Na adsorption at the interface of the organic lining could explain the relative increase of the trough compared to peak height with increasing salinity. Still, Na/Ca troughs appear unrelated to large organic structures (>100 nm) and future studies should focus on the structural position and crystallographic coordination of Na.

Organic linings can also introduce lattice strain, which could explain enhanced and coupled element incorporation around the organic linings. Recent studies have suggested that D_Sr_ increases with increasing Mg content due to the effect of Mg-induced lattice strain, between species^45^ and between specimens of *A. lessonii*^73^. The relationship between Mg/Ca and D_Sr_ in hyaline species^28,45^ and in *A. lessonii* specimens^8,73^ closely resembles the relationship for inorganically precipitated calcite^68^. As Mg disrupts the calcite crystal structure by introducing lattice strain, its incorporation should also affect the partition coefficients of other elements incorporated into the calcite structure, such as Na^65^ and Sr^75^. A Mg-induced lattice strain effect could hence also explain co-varying Sr and Na with Mg bands in *A. lessonii* similar to inorganic precipitation studies. At low Mg levels it is unlikely that Sr and Na will be significantly impacted by Mg-induced lattice strain. Hence, the 8–10 times steeper inter-element slope for *A. tepida*, at the intra-shell and inter-specimen level, hints at a higher partition coefficient. However, inorganic precipitation experiments are required to quantify the effect of increasing Mg-content on Na incorporation, as well as how coupled effects of D_Me_ due to lattice strain may be altered by the formation of precursor phases such as vaterite and ACC.

#### Modes of ion transport to the site of calcification

Abiotic precipitation experiments from artificial seawater result in calcites with high Mg/Ca ratios compared to those in foraminifera. The organism hence must be able to lower Mg/Ca ratios relative to seawater Mg/Ca. Currently, the mechanisms by which Mg and Ca (and other ions) reach the site of calcification in foraminifera is heavily debated. Below, we discuss how the proposed mechanisms could or could not explain our observations.

The two transport models that have gained relatively high attention are the ‘trans-membrane transport’ (TMT) model^40^ and seawater vacuolization model^37^. Both models rely on selective ion transport to create the low-Mg/Ca solution from which calcification commences in foraminifera^38^. In the TMT model, selective Ca-transport to the site of calcification (SOC) occurs in combination with ‘passive transport’ of seawater leaking from the environment into the SOC. Alternatively, in the second model vacuolized seawater undergoes selective ion (i.e. Mg^2+^) removal before the release of a low-Mg/Ca fluid into the SOC^26,37^ (Erez, 2003; Bentov and Erez, 2006). In addition to these two biomineralization models, ions (and their isotopes) may undergo additional partitioning and fractionation due to processes operating during calcite precipitation (e.g. Rayleigh fractionation^72^ and lattice strain effects^76^), which will be discussed later. Our results cannot distinguish between these types of ion transport, but both TMT and vacuolization are used to describe the change in ion supply over time and its potential in explaining element banding.

In the both biomineralization models based on ion transport, variation of El/Ca within the shell wall could result from changes in the elemental composition of the SOC (e.g. the El/Ca ratio) and/or changes in the inorganic partition coefficients (e.g. D_Me_), as expressed by the following equation:

$$El/C{a}_{(t)}={D}_{El}\times \frac{E{l}_{SOC}}{C{a}_{SOC}}$$

D_El_ is often estimated from inorganic precipitation experiments in which precipitation usually proceeds through classical crystal growth^68,77^. Element incorporation in these studies is shown to be affected by pH of the seawater, which should therefore be taken into account when translating inorganic partition coefficient to partitioning in the SOC during calcification^37,39,40,45,72^.

Species with very low average Mg/Ca, like *Ammonia tepida*, but also planktic species often used in paleo-applications, may be characterized by a SOC that is well-separated from the surrounding seawater^78^. Ions necessary for calcification may enter the calcifying fluid by selective inward Ca^2+^ trans-membrane transport (TMT) enclosing the site of calcification^26,37,40,43^ or by Ca^2+^ from vacuolized seawater^37^. Both the increase of the pH (due to proton pumping) and the higher Ca^2+^ concentration at the SOC will facilitate CaCO_3_ nucleation and growth by increasing the saturation of the fluid present with respect to calcite. Inward Ca^2+^-transport is then assumed to be the dominant source of Ca in the SOC and hence determines (the low) El/Ca of the fluid from which the calcite precipitates. Foraminiferal species precipitating their calcite from an open, more seawater-like, SOC, or often replenished biomineralization space^72^, will show El/Ca values close to ratios reported in inorganic precipitation experiments from seawater. A fluid with a El/Ca resembling seawater might derive ions from seawater vacuoles, which have been observed in higher Mg species such as *Amphistegina lobifera*^76^. Higher fractions of unmodified seawater and lower Ca^2+^-transport (both increasing the El/Ca ratio towards seawater El/Ca) could both explain why all El/Ca ratios measured here are higher in the intermediate Mg species *A. lessonii* compared to low-Mg species *A. tepida*.

Variability in the relative contributions of selectively transported Ca^2+^ and seawater/modified vacuoles might hence not only explain differences in El/Ca (co-)variability at the inter-species level^40^, but also explain differences in El/Ca between specimens. Variable Ca^2+^ transport during the chamber calcification, could explain intra-specimen El/Ca variability (e.g. element banding) if Ca^2+^ levels are in- and decreasing during chamber formation. Alternatively, hypothesized outward Mg^2+^-transport^26^ would also be reflected in El/Ca banding perpendicular to the growth direction of the shell. As selective removal of Mg^2+ ^^26,37^ would have a dominant effect on Mg-banding and only a limited effect on banding of other elements (with possibly the exception of Sr and Na in high-Mg species) it is more likely that Ca-pumping or passive transport variability is responsible for the synchronous banding in all metals.

Ca-transport models may thus explain the observed inter-elemental correlations within the shell (Table 3, Fig. S6), between specimens^8^ and between hyaline foraminifera^28,29,45,52^. This is further corroborated by the observation that the inter-element trend for *A. lessonii* specimens and for hyaline foraminiferal species approximately falls into the line between El/Ca = zero and El/Ca_seawater_ ratios times the inorganic D_El_’s (albeit that the intercept for Na and Sr is >0, which could be explained by the additional impact of adsorbed cations at the organic linings).

The slopes of the intra-shell Mg/Ca-Na/Ca and Mg/Ca-Sr/Ca relationships are respectively 8 and 10 times higher for *A. tepida* than for *A. lessonii* (Fig. S6; Table 3). The same increase in slopes is observed for the inter-specimen relationship between these elements^8^. Hence, our NanoSIMS observations on intra-specimen combined with inter-specimen inter-element correlations^8^ suggest that there must be a parameter affecting element incorporation that differs between the two species studied here. This could be related to changes in the partition coefficient D_Na_, between species (and potentially, over time), which then should be about 8 times higher in *A. tepida* compared to *A. lessonii*. The partition coefficient, in turn, could be affected by calcite growth rates, which is likely affected by the pH and [Ca^2+^] at the SOC^68,77^. A difference in precipitation rates between species could, for example, explain a higher D_Na_ in *A. tepida* compared to *A. lessonii*^79^, although this hypothesis requires future testing. Additionally, a higher Mg^2+^ concentration in *A. lessonii* could slow down precipitation rates by increasing mineral solubility^80^.

Phase transformations during calcite precipitation are another variable affecting element partitioning. Recently, a vaterite precursor phase has been identified in several foraminiferal species^81^, implying inorganic partition coefficients might have to be reassessed since the partition coefficients in step-wise calcite growth may differ from partitioning through a vaterite precursor. This process has been suggested to also allow for low Mg values without the need of complex ion-transport models, by means of (double) fractionation against Mg through phase-transitions from ACC to vaterite (not observed yet but hypothesized) and vaterite to calcite^81^. It could be imagined that species with higher Mg, such as *A. lessonii*, a direct phase transformation from ACC to calcite, due to the presence of Mg, explains the difference between low and slightly elevated Mg levels (hence through a single or double fractionation step). Irrespective of the CaCO_3_ phase(s) involved, ion incorporation may be also be affected by Rayleigh fractionation during the formation of a chamber, which could explain the increase in El/Ca towards the end of the lamella as observed in *A. lessonii* specimens (Fig. 3).

If calcite precipitation takes place in a (semi-) enclosed environment, differences between ions in incorporation rates result in a change in the ratios between these ions in the SOC due to Rayleigh fractionation effects^26,37,38,43,54,78^. This, in turn increasingly affects the element composition of the layers precipitated subsequently, resulting in increasing or decreasing element concentrations over time^39,72,76^. Such a process could additionally affect element concentrations and if the mode of ion transport into the SOC (either by TMT or vacuoles) changes over time, would give rise to a pattern of alternating high- and low El/Ca bands. In theory, the combination of all processes mentioned above operate in concert and are likely required to fully explain element banding and its variability across species.

## Conclusions

Despite their different average El/Ca ratios, specimens of both the benthic foraminifers *Amphistegina lessonii* and *Ammonia tepida* have chamber walls that show clear element banding patterns for Mg, Na, Sr, K, S, P and N. Peak locations overlap for all elements measured here and coincide with the location of the organic linings separating the calcite lamellae of the shell walls. Peak widths of S and P, associated to the organic linings, are significantly smaller than El/Ca peak widths, especially in *A. lessonii*. The 10-fold difference in average Mg/Ca (~33 mmol/mol in *A. lessonii* versus ~3 mmol/mol in *A. tepida*) corresponds to a ~10 fold increase in peak and trough height of Mg/Ca in *A. lessonii* compared to *A. tepida*. Furthermore, we show that the entire within-shell wall El/Ca-distribution is raised in specimens with higher El/Ca average values. Hence, peak and trough heights show a proportional (in *A. lessonii*) or linear (in *A. tepida*) relationship between specimens, whether grown in different conditions or the same conditions and reflecting inter-specimen variability in El/Ca. This observation enhances confidence in proxies based on element incorporation in foraminiferal calcite, as the ratio between low- and high-concentration bands is not affected by the environmental condition. The observed 8-fold increase in Mg/Ca-Na/Ca slopes between *A. tepida* and *A. lessonii* intra-specimen trends in our NanoSIMS maps and at the inter-specimen level^8^ hint at a difference in the calcification pathway between these species.

## Supplementary information

Supplement

## Data Availability

The data on which our results are based can be found through the following doi:10.4121/uuid:9a0774a1-39d1-4fde-9e83-b64e9f81e4e0.
