# Supplementary material for: Element banding and organic linings within chamber walls of two benthic foraminifera
Source: Sci Rep. 2019 Mar 5;9:3598. doi: 10.1038/s41598-019-40298-y (PMC6400897; doi:10.1038/s41598-019-40298-y)
Supplement: Supplementary file 1 — Supplement [file 41598_2019_40298_MOESM1_ESM.pdf]

Supplements to: *Element banding and organic linings within chamber walls of two benthic foraminifera*

E. Geerken, L.J. de Nooijer, A. Roepert, L. Polerecky, H. E. King, G.J. Reichart

Supplementary figure S1.

Alignment of an external and NanoSIMS image using a new interactive tool in Look@NanoSIMS. First, the external image (here an SEM image) and the NanoSIMS image (here the accumulated image of Ca+ ion counts) are loaded into the tool (panel A) and their overlay is displayed (panel B). Subsequently, keyboard is used to change in small increments the relative angle, displacement and magnification of one image against the other while observing their overlay. These interactive steps are repeated until a visually satisfactory alignment is achieved (panel C).

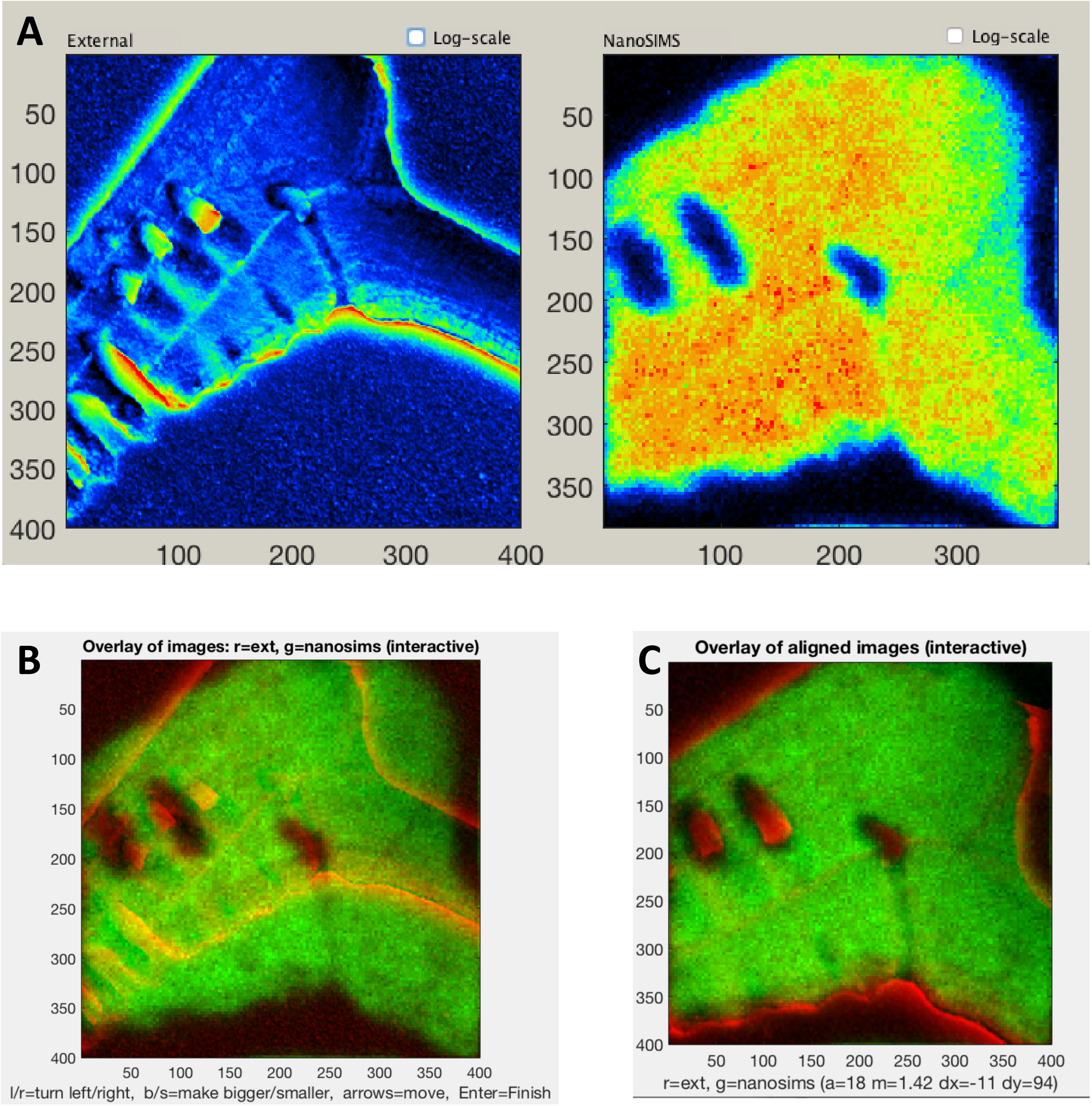

Supplementary Figure S2.

Panel A: Maximum, mean and minimum count ratios for Mg/Ca, Na/Ca and Sr/Ca based on NanoSIMS maps obtained for *A. tepida* and *A. lessonii* (in red the 95th percentile, in green the mean and in blue the 5th percentile ion counts of the lateral profiles), versus individual average LA-ICP-MS El/Ca values, with the 95% confidence bounds for the fitted coefficients in dashed lines. The relationship between the mean count ratios and LA-ICP-MS El/Ca values is used for the calibration of ion counts to molar ratios, see table 2 and suppl. table 1 for regression results. Panel B: distribution plots for Na/Ca, Mg/Ca, Sr/Ca and K/Ca, showing the density distributions of El/Ca values observed within the lateral profiles, color-coded per environmental condition. Dashed/ non-dashed lines indicate whether lateral profiles are from different maps on the same specimen. The top row shows the *A. lessonii* specimens from the temperature experiment, the middle row specimens of *A. lessonii* from the salinity experiment and bottom row those of *A. tepida* from the salinity experiment. The entire intra-shell spatial distributions shift for specimens grown in different conditions. Within temperature and salinity conditions, different specimens also show a shift in the density distributions, whereas in most cases, density distributions of different maps on the same specimen are similar.

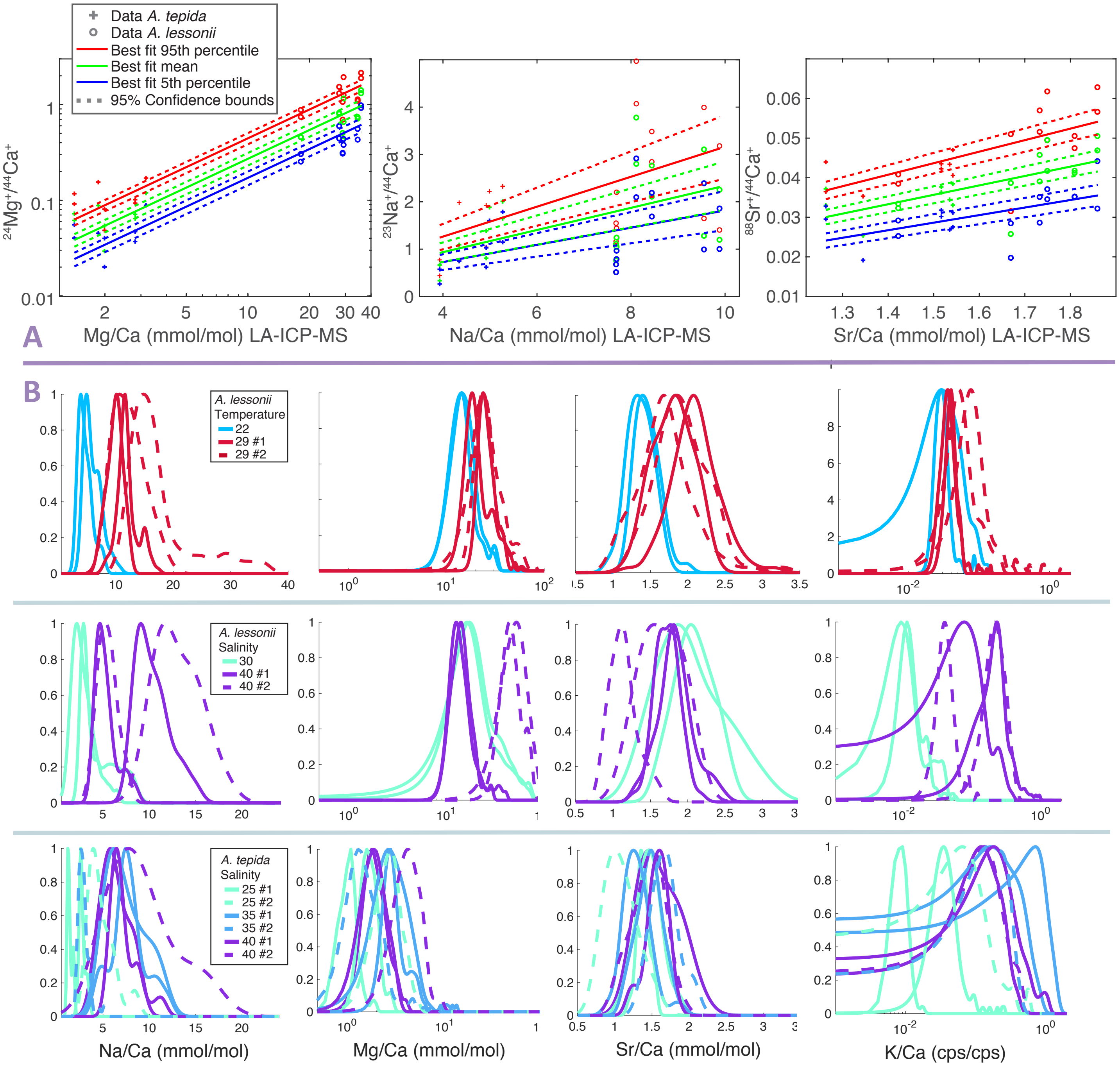

Supplementary Figure S3.

Examples of representative NanoSIMS maps of P, CN and S (ratio to O) for specimens of *A. lessonii* (panel A) and *A. tepida* (panel B). Lateral profiles, indicated by a white arrow from the inside to the outside of the shell, showing clear peaks at the position of the organic linings separating the calcite lamella (fig. 1). Color bars are the same for each element to enhance comparability between maps. All scale bars are 3 µm.

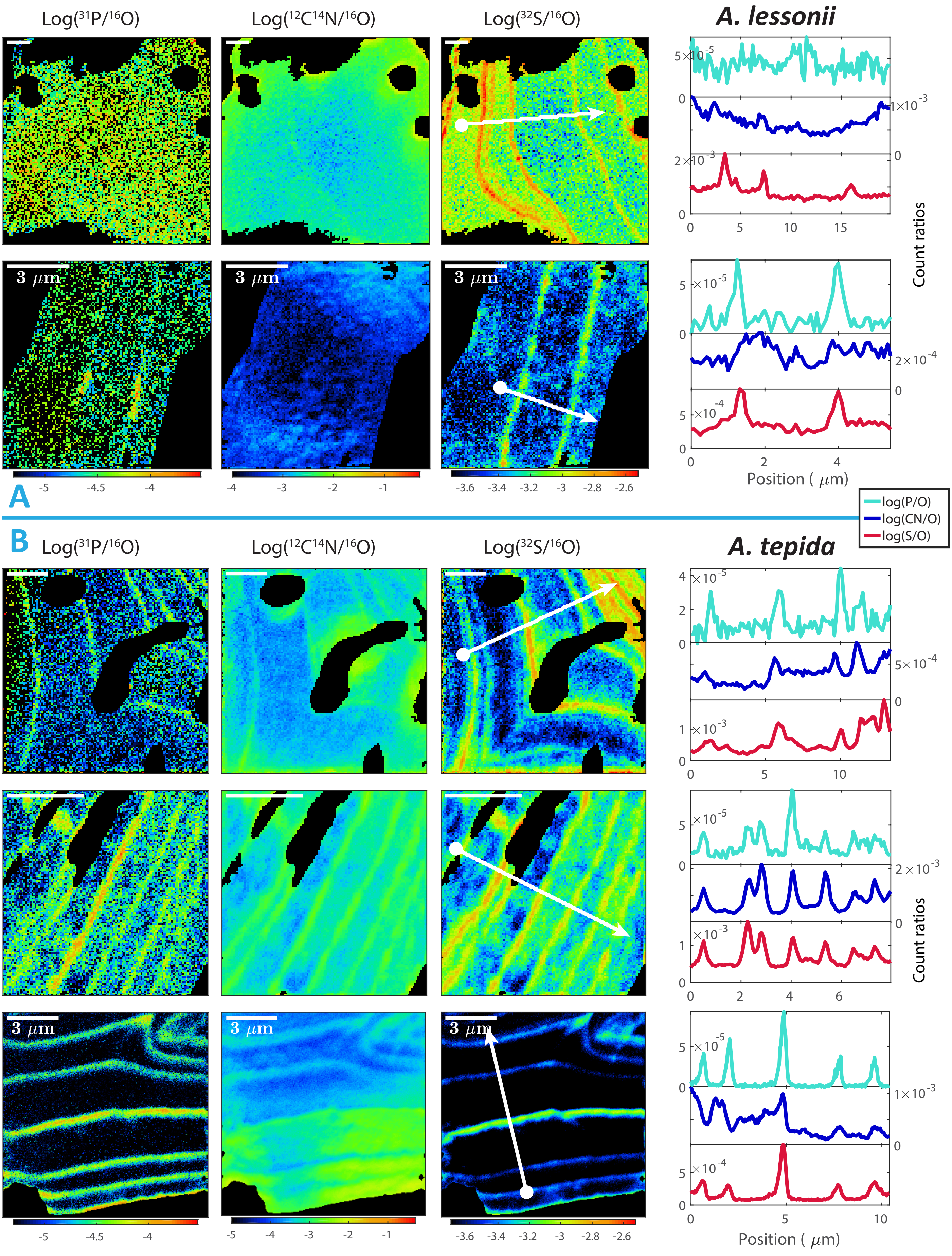

Supplementary Figure S4.

Comparison between the sample topography obtained by AFM after the NanoSIMS measurements and the NanoSIMS-derived secondary ion counts and ratios (El/Ca). Shown are aligned images as well as lateral profiles along directions indicated by arrows in the images. Top right panel shows that the surface has a raising edge between positions of about 6−7 µm and a falling edge between positions of about 10−11 µm. If the distinct Na/Ca and Mg/Ca peaks observed at the raising edge (position ~6 µm) were an artifact due to surface topography, we would expect a similarly distinct peak at the falling edge, which is not the case. Second row shows a similar situation in a different sample. The lower two rows show that distinct El/Ca peaks in *Ammonia tepida* are of similar height as those in *Amphistegina lessonii* even though the variability of the surface height is relatively low in these samples.

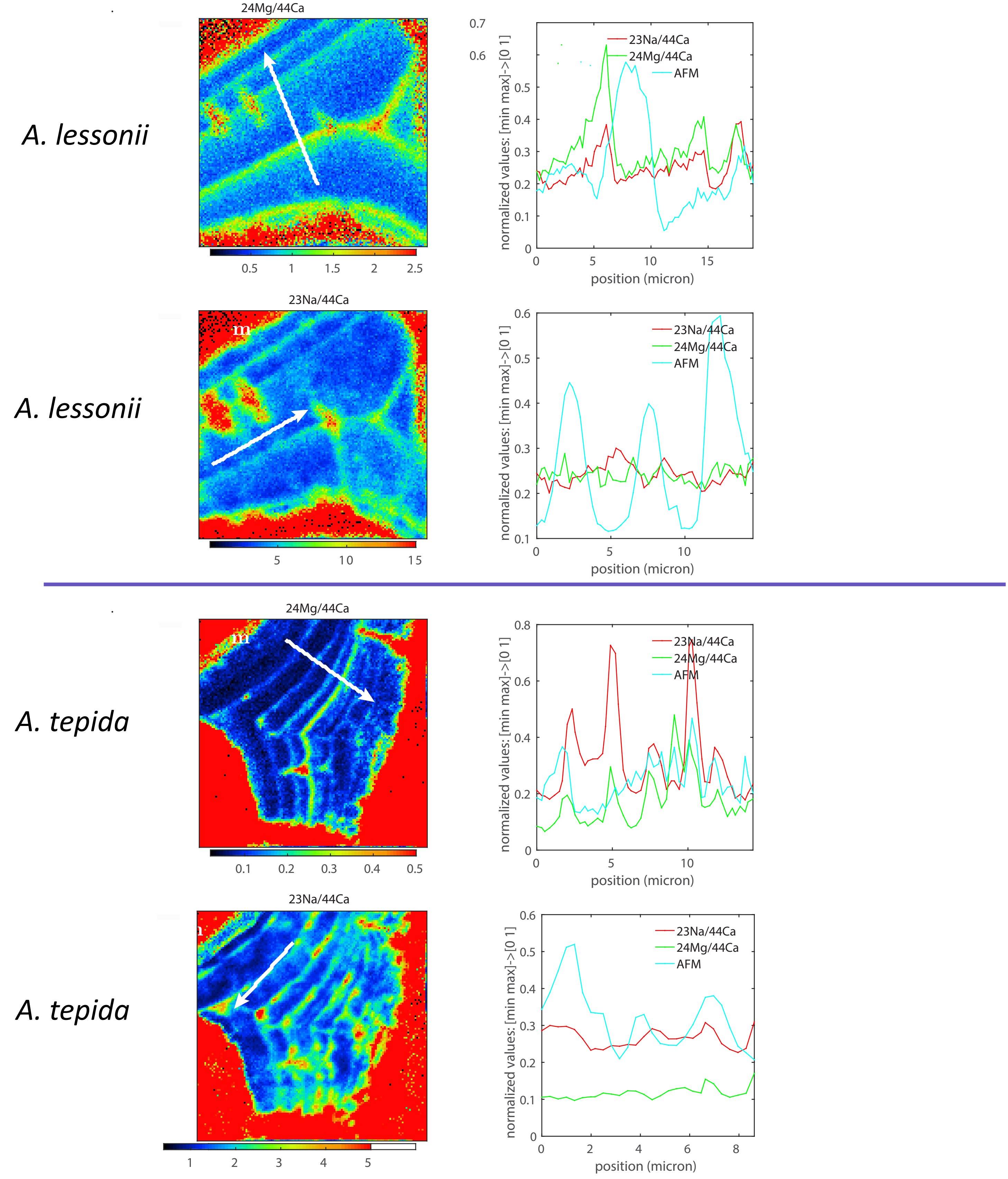

Supplementary Figure S5.

A). Convolutions (in green) of a top-hat function (red) of 270 nm (average 'organic' El/O peak width as measured with the Cs-source) and Gaussian functions (blue) with a FWHM (full width at half maximum) based on adjusted Python script by Branson et al. (2016). The Gaussian FWHM is based on the nominal O-beam size expected with our settings as measured by Cameca, expected to be ~300nm with a maximum of 500 nm, which is converted to FWHM as 355 (solid lines) and 640 nm (dashed lines). B) Boxplots showing the FWHM peak width distributions of *A. lessonii* and *A. tepida* El/Ca (Sr/Ca, Mg/Ca and Na/Ca) and P/O peak width distributions, measured with the Cs-source with a smaller nominal beam size (100nm), adjusted for the O-beam size effect, with 355 nm being the expected beam size and 640 nm the maximum beam size. Letters above the boxplot show whether distributions are significantly different. For the expected beam size of 355 nm, both *A. lessonii* and *A. tepida* peak width distributions are significantly different from the beam-size corrected P/O peak widths distributions. For the maximum beam size of 640 nm, only *A. lessonii* peak widths are significantly different from the beam-size corrected P/O peak widths distribution.

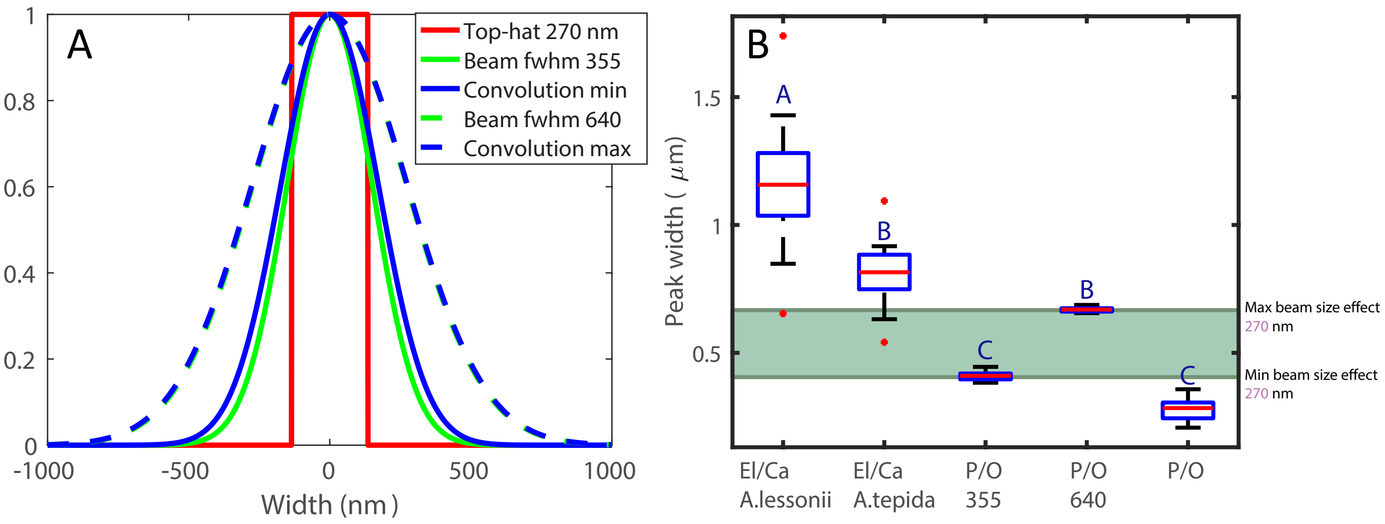

Supplementary Figure S6.

Scatterplots of the lateral profiles drawn from NanoSIMS count ratio maps, with orthogonal regression lines showing the relationship between elements within the shell-wall. Lateral profiles from all specimens, grown in different conditions (color-coded), are significantly correlated for Mg/Ca versus Na/Ca (A), Mg/Ca versus Sr/Ca (B) and K/Ca versus Na/Ca (C) for *A. tepida* (left) and *A. lessonii* (middle=salinity, right=temperature). See table 2 for regression results.

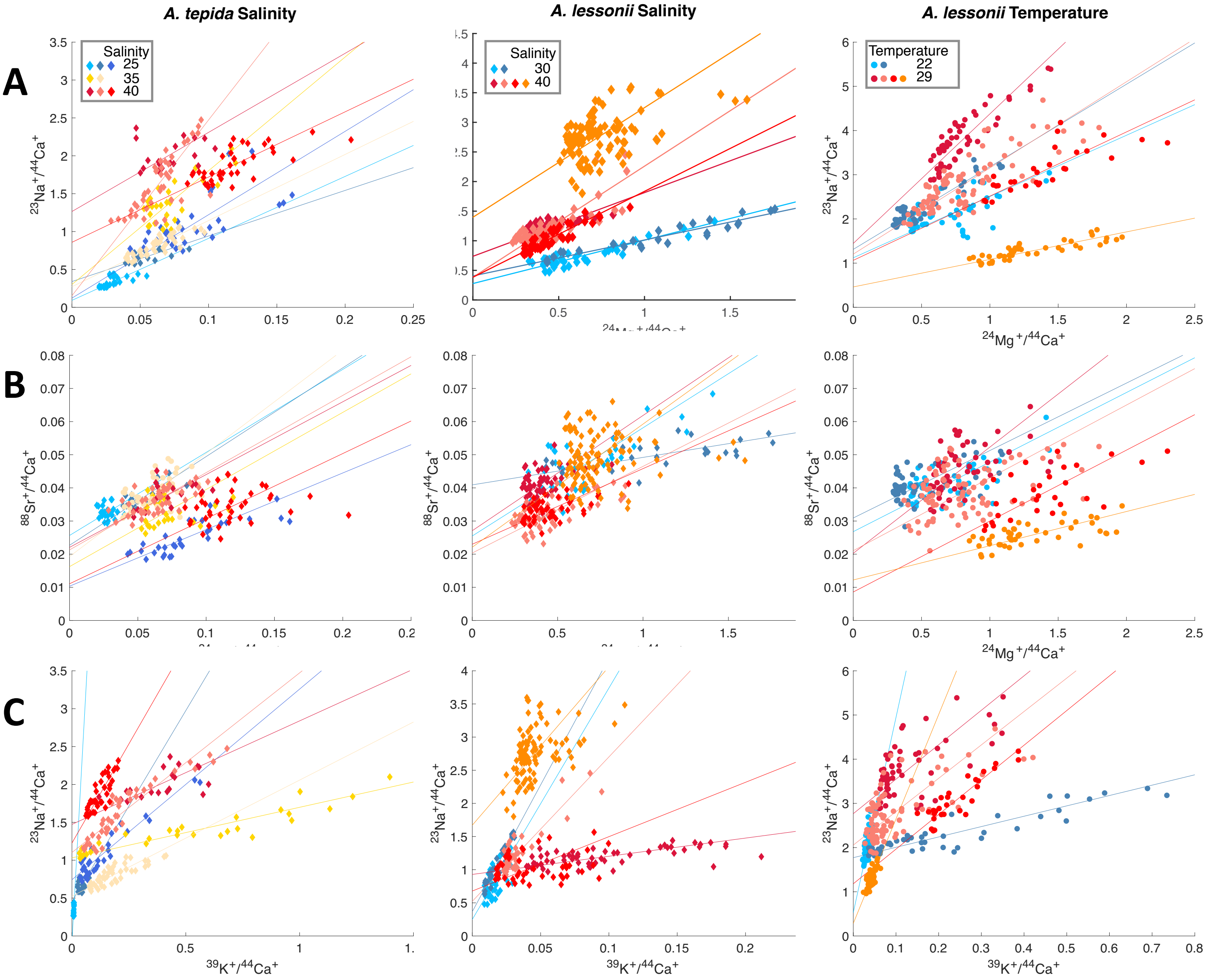

Supplementary table 1. Linear regression results ($El/Ca_{ioncounts}$=slope*$El/Ca_{LA\text{-}ICP\text{-}MS}$+ intercept) for the maximum (95th percentile) and minimum (5th percentile) of the NanoSIMS lateral profiles versus individual LA-ICP-MS values. To account for the order of magnitude difference in the average Mg/Ca between *A. tepida* and *A. lessonii*. Both the NanoSIMS ion counts and the LA-ICP-MS-derived concentrations were log-transformed. Regression analysis for the concentrations and ion/ion counts of Na/Ca and Sr/Ca were forced through zero.

El/Ca	Percentile	Slope	SE_slope	tStat	Intercept	R^2	p-Value
**log(Mg/Ca)**	95%	0.94	0.06	15.34	-1.29	0.93	8.87E-12
	5%	0.89	0.06	14.18	-1.65	0.92	3.28E-11
**Na/Ca**	95%	3.16E-01	3.21E-02	9.85		0.28	6.67E-09
	5%	1.82E-01	1.97E-02	9.20		0.26	1.99E-08
**Sr/Ca**	95%	2.91E-02	8.30E-04	35.08		0.46	9.72E-19
	5%	1.91E-02	6.84E-04	27.89		0.35	7.03E-17

Supplementary table 2. Linear regression results (Peak height=slope × trough height + intercept) for the relationships between peak and trough heights of the lateral profiles. For *A. lessonii,* the linear regression model on the log-transformed peak and trough values gives the best fit. For *A. tepida,* both regressions result in a similar R^2.

Species	Data-type	SE_slope	tStat	Intercept	R^2	p-Value
A. lessonii	Normal	2.17	15.21	-0.05	0.87	9.87E-17
	Log-Log	1.12	32.58	0.19	0.97	3.18E-27
A. tepida	Normal	1.40	10.29	0.72	0.83	7.22E-10
	Log-Log	0.96	11.02	0.09	0.85	2.01E-10
